# Rapidly Progressive Mucoepidermoid Carcinoma in an Adult Patient: A Case Report

**DOI:** 10.7759/cureus.94720

**Published:** 2025-10-16

**Authors:** Reshma Hammannavar, Chaitanya Birole, Vaishnavi Bhavsar, Harshal Patil, Mayur Lanje, Seema Gupta

**Affiliations:** 1 Department of Oral Surgery, Jawahar Medical Foundation's Annasaheb Chudaman Patil Memorial Dental College, Dhule, IND; 2 Department of Oral and Maxillofacial Surgery, Jawahar Medical Foundation's Annasaheb Chudaman Patil Memorial Dental College, Dhule, IND; 3 Department of Orthodontics, Kothiwal Dental College and Research Centre, Moradabad, IND

**Keywords:** mucoepidermoid carcinoma, neck dissection, salivary gland malignancy, sublingual gland, submandibular gland

## Abstract

Among salivary gland malignancies, mucoepidermoid carcinoma (MEC) often arises in the parotid gland and is less commonly found in the submandibular gland. This case report describes a 53-year-old Asian male patient presenting with a two-month history of insidious left submandibular swelling that progressed to pain in the preceding 10 days. Clinical examination revealed a firm, 3.5 cm x 2.5 cm mass with sublingual extension. Fine-needle aspiration cytology suggested salivary gland malignancy, confirmed by histopathological and immunohistochemistry analysis. To optimize access, surgical management involved en bloc resection of the left submandibular and sublingual glands with 1.5 cm margins, modified radical neck dissection (type III), and extraction of the left lower teeth from the lateral incisor to the second premolar. Histopathology confirmed intermediate-grade MEC with clear margins and focal perineural invasion. At follow-up, the patient remained disease-free with excellent functional outcomes, avoiding adjuvant radiotherapy. This case underscores the efficacy of multimodal diagnosis and tailored surgery for submandibular MEC, emphasizing the need for vigilant surveillance.

## Introduction

Salivary gland tumors represent a diverse group of neoplasms, with malignant variants accounting for 6.02 per 100,000 population as the European-age standardized incidence rate, posing significant diagnostic and therapeutic challenges owing to their histological heterogeneity and potential for regional metastasis [1]. Among these, mucoepidermoid carcinoma (MEC) is the second most prevalent malignant salivary gland tumor, comprising 10-15% of all salivary gland neoplasms and nearly one-third of all salivary malignancies [2,3].

MEC is characterized by a mixture of mucous, intermediate, and epidermoid cells and exhibits a wide spectrum of biological behavior, ranging from indolent low-grade lesions to aggressive high-grade tumors with poor prognosis [4,5]. The parotid gland is the most frequently affected site, with over 50% of cases, followed by the submandibular gland (8-10%), sublingual gland (rare), and minor salivary glands of the oral cavity [1-4]. Epidemiologically, MEC predominantly manifests in adults aged 35-65 years, with a slight female predominance, although it is the most common salivary malignancy in pediatric populations, where up to 35% of salivary neoplasms are malignant [5].

Clinically, MEC often presents as a painless, slowly enlarging mass, although symptoms such as pain, facial nerve involvement, and trismus may emerge in advanced or high-grade cases, particularly when involving intraoral extensions [6]. Submandibular gland involvement, as in the present case, is particularly noteworthy because of its proximity to critical neurovascular structures and higher propensity for locoregional spread than parotid tumors, with lymph node metastasis occurring in 20-50% of cases [7]. The integration of molecular assays with histological attributes is essential for the complex differential diagnosis of MEC. A thorough comprehension of the characteristics of other lesions that may mimic MEC is imperative, especially in the context of adenosquamous carcinoma and various tumors that exhibit mucinous components [8]. Histopathological grading, based on cystic components, neural invasion, mitoses, and necrosis, guides prognosis and management, with low-grade tumors showing five-year survival rates exceeding 90%, whereas high-grade variants drop to 30-50% [7].

Treatment paradigms emphasize multimodal approaches tailored to grades and stages. Surgical resection remains the cornerstone, aiming for wide local excision with negative margins, often coupled with neck dissection. Adjuvant radiotherapy is indicated for high-grade tumors, close margins, or extracapsular nodal spread, while chemotherapy is reserved for metastatic or recurrent disease with limited efficacy [9]. Despite advances, challenges persist in submandibular MEC, including functional morbidity from gland excision and dissection-related complications, such as marginal mandibular nerve injury.

This case report highlights a 53-year-old male patient with submandibular and sublingual gland involvement with MEC, underscoring the insidious presentation mimicking benign pathology and the efficacy of comprehensive surgical intervention. By detailing the diagnostic journey, multidisciplinary management, and favorable short-term outcomes, we aimed to contribute to the literature on rare salivary malignancies, emphasizing early detection and aggressive yet conservative strategies to optimize survival and quality of life in non-parotid sites.

## Case presentation

A 53-year-old Asian man, a non-smoker with no significant medical history, presented to the Department of Oral and Maxillofacial Surgery at Jawahar Medical Foundation’s Annasaheb Chudaman Patil Memorial Dental College, Dhule, India, on January 15, 2024. He reported no drug allergies, comorbidities, or family history of malignancy. His dental history included routine checkups without prior intervention in the affected region. He complained of gradually progressive swelling in the left lower jaw near the back teeth, noticed two months prior. The swelling was insidious, initially asymptomatic, and had grown to approximately 3.5 cm x 2.5 cm without trauma or infection. Ten days before the consultation, he developed intermittent dull pain in the left submandibular region, worsened by chewing, and relieved by analgesics. No fever, weight loss, dysphagia, odynophagia, or salivary discharge was noted. Extraoral palpation revealed a firm, non-tender, mobile mass in the left submandibular triangle without changes in skin characteristics or cervical lymphadenopathy.

**Figure 1:**
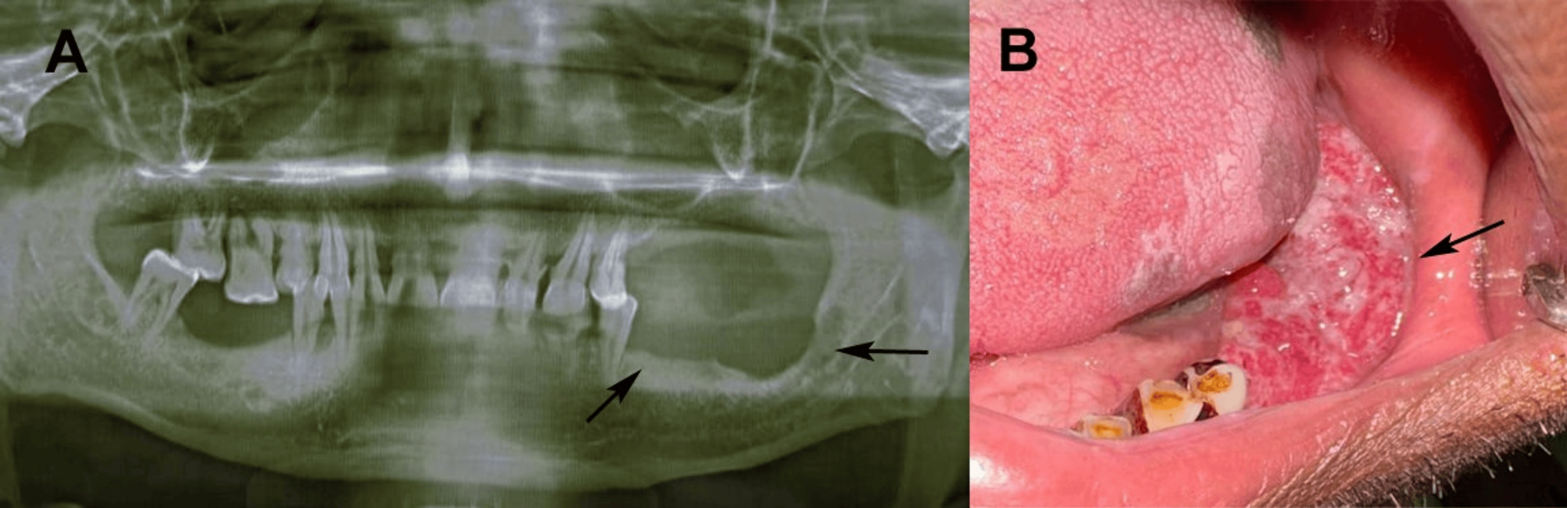
(A) Panoramic radiograph (OPG) showing alveolar ridge erosion from the premolar to the third molar area (black arrow) and (B) intraoral examination showing non-homogeneous irregular exophytic growth extended from the lower left second premolar area to third molar area. Original patient's images, used with patient's consent for publication.

On examination, a solitary, well-defined, oval-shaped swelling (3.5 cm x 2.5 cm) was noted in the left submandibular region, firm, non-fluctuant, and mildly tender on deep palpation, with restricted mobility due to deeper tissue adherence. No facial asymmetry, trismus, or cranial nerve deficit was observed. The left submandibular gland was asymmetrically enlarged, with no suppurative inflammation. Intraorally, a swelling in the left lingual vestibule extending from the lower left second premolar (tooth 35) to the retromolar region presented as a smooth, dome-shaped, erythematous mucosa-covered mass with intact epithelium and no ulceration or bleeding. The mucosa was tense, but non-punctate. Teeth 32-35 (lower left lateral incisor to lower left second premolar) were vital, non-carious, and non-mobile, with no periodontal pockets or abscesses. The floor of the mouth was elevated to the left, suggesting sublingual extension, but the tongue and oropharynx were unremarkable. An orthopantomogram (OPG) showed vague radiolucency in the left mandibular body adjacent to the lesion, without cortical erosion (Figures 1A, 1B). Contrast-enhanced computed tomography (CT) of the head and neck revealed a well-circumscribed soft-tissue mass involving the left submandibular and sublingual glands, abutting but not infiltrating the mylohyoid muscle. No mandibular cortical erosion, perineural spread, or distant nodal enlargement beyond level II was observed. These findings guided the extent of resection and neck dissection planning.

The preoperative workup included a complete blood count (CBC), liver function test, kidney function test, serological tests for Epstein-Barr virus, human immunodeficiency virus, and Hepatitis B virus, and tumor markers, including carcinoembryonic antigen, and squamous cell carcinoma antigen (Table 1).

**Table 1:** Summary of patient laboratory investigations, with all measured values falling within normal limits. g/dL: Grams per deciliter, μL: Microliter, mg/dL: Milligrams per deciliter; U/L: Units per liter; ng/mL: Nanograms per milliliter.

Test	Test value	Normal limits
Hemoglobin (Hb)	13.8 g/dL	Male: 13.5 - 17.5 g/dL, Female: 12.0 - 15.5 g/dL
Leukocyte	7,200/μL	4,500 - 11,000 /μL
Platelets	280,000/μL	150,000 - 450,000 /μL
Creatinine	0.9 mg/dL	~0.7 - 1.3 mg/dL
Bilurubin	0.7 mg/dL	0.1 - 1.2 mg/dL
Alanine aminotransferase (ALT)	25 U/L	Male: 7 - 55 U/L, Female: 7 - 45 U/L
Aspartate aminotransferase (AST)	28 U/L	10 - 40 U/L
Prothrombin time	12 seconds	11 - 13.5 seconds
Epstein-Barr virus	Negative	Negative
Human immunodeficiency virus	Negative	Negative
Hepatitis B	Negative	Negative
Carcinoembryonic antigen	1.2 ng/mL	Non-smokers: ≤3.0 ng/mL, Smokers: ≤5.0 ng/mL
Squamous cell carcinoma antigen	0.5 ng/mL	<1.5 - 2.0 ng/mL

Fine-needle aspiration cytology (FNAC) was performed under aseptic conditions using a 22-gauge needle attached to a 10 mL disposable syringe, inserted intraorally through the left lingual vestibule into the swelling. Approximately 1 mL of thick, straw-colored mucoid material was aspirated and immediately smeared onto glass slides. The FNAC in this case was performed under ultrasound guidance to ensure accurate sampling from the solid component of the lesion and minimize cystic contamination. Cytological interpretation was classified according to the Milan System for Reporting Salivary Gland Cytopathology, corresponding to Category V (suspicious for malignancy), consistent with low-to-intermediate grade mucoepidermoid carcinoma [10]. The smears were fixed in 95% ethanol and stained with hematoxylin and eosin (H&E), Papanicolaou, and May-Grünwald-Giemsa stains for cytologic evaluation. Microscopy revealed a triphasic cell population composed of (i) polygonal squamous (epidermoid) cells with moderate nuclear pleomorphism and distinct cell borders, (ii) mucous cells containing intracytoplasmic mucin vacuoles forming glandular clusters, and (iii) intermediate cells with clear cytoplasm and centrally placed nuclei. The background showed abundant mucin and scattered lymphocytes, without necrosis or mitotic activity. The cytologic impression favored low- to intermediate-grade MEC. These findings, consistent with the clinical picture of a firm, painless submandibular swelling, prompted a recommendation for en bloc surgical excision with neck dissection for definitive management and histopathologic confirmation.

**Figure 2:**
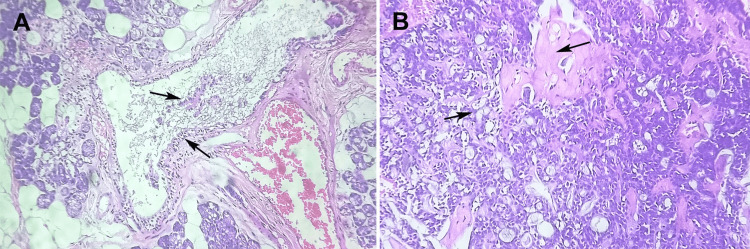
(A) Hematoxylin and eosin-stained section at 40X magnification showed a cystic area lined with mucous and epidermoid cells and (B) hematoxylin and eosin-stained section at 40X magnification showed solid nests composed of intermediate and epidermoid cells. Original image of hematoxylin and eosin stained section of excised lesion at 40X magnification.

Following the incisional biopsy, histopathology with immunohistochemistry (IHC) confirmed intermediate-grade MEC. H&E-stained sections showed a triphasic morphology: epidermoid cell nests, intermediate cells, and mucinous cells in cystic spaces (Figures 2A, 2B). Mucicarmine staining was performed to confirm the presence of mucin-producing cells, while PAS-D staining was not conducted. The immunohistochemical panel included cytokeratin AE1/AE3, CK7, p63, MUC5AC, S100, SMA, and Ki-67. p63 was used for evaluating squamous differentiation, as p40 was unavailable at the time of testing. The tumor was graded according to the Brandwein-Gensler grading system, based on parameters including mitotic activity, necrosis, and perineural invasion [11]. The tumor exhibited mucicarmine-positive mucinous cells. Immunohistochemically, the epithelial cells were positive for AE1/AE3, CK7, and p63, while S100 and SMA were negative. MUC5AC confirmed mucinous differentiation, and the Ki-67 proliferation index was approximately 15%. According to the Brandwein-Gensler criteria, considering moderate mitotic activity, focal necrosis, and perineural invasion, the tumor was classified as intermediate grade.

Based on clinical, FNAC, and IHC findings, a diagnosis of intermediate-grade MEC of the left submandibular and sublingual glands with N1 nodal disease (T2N1M0, Stage III per the American Joint Committee on Cancer (AJCC) 8th edition) [11]. The T2N1M0 classification indicates a tumor 2-4 cm in size (T2), involvement of a single ipsilateral lymph node (N1), and no distant metastases (M0). A multidisciplinary tumor board recommended surgical resection with neck dissection. Preoperative optimization included dental prophylaxis, nutritional assessment (BMI 24 kg/m²), and counseling on risks such as infection, hematoma, nerve injury, and recurrence.

**Figure 3:**
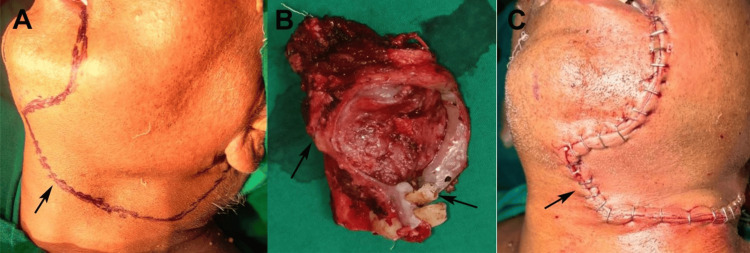
(A) A modified Blair incision's marking, (B) excised specimen with margins and teeth (from the lower left lateral incisor to lower left second premolar), and (C) suturing with Vicryl 3.0. Original patient's images, used with patient's consent for publication.

Surgery was performed under general anesthesia via nasotracheal intubation on February 5, 2024. A modified Blair incision facilitated en bloc excision of the left submandibular and sublingual glands (Figure 3A), including the adherent floor of the mouth mucosa and the mylohyoid muscle, preserving the lingual nerve (Figure 3B). Mandibular involvement was not observed. Modified radical neck dissection (MRND) type III (levels I-V, sparing the internal jugular vein, spinal accessory nerve, and sternocleidomastoid) yielded 28 lymph nodes. Of the 28 cervical lymph nodes dissected, two nodes at Level II were positive for metastatic deposits, measuring 0.8 cm and 1.1 cm, respectively. No extranodal extension (ENE) was identified. Teeth 32-35 were extracted to optimize the access and prevent fistula formation. An MRND included nodal levels I-V, to achieve oncologic clearance given the radiologically enlarged Level II nodes and the drainage pattern of the submandibular region. Levels IV-V were included to eliminate the possibility of skip metastasis. Contralateral neck evaluation was not indicated due to the absence of clinical or radiologic lymphadenopathy.

The resection margins were histologically free of tumor, ranging from 1 mm (deep margin) to 3 mm (superficial margin). At our institution, a ≥1 mm tumor-free margin is considered adequate for head and neck malignancies. Intraoperatively, the mandibular periosteum appeared intact, and no bone involvement was evident. Frozen section analysis or bone biopsy was therefore not indicated. Hemostasis was performed using bipolar cautery with 150 mL of blood loss. The defect was closed with Vicryl 3-0 and Monocryl 4-0, with a 10-Fr drain. The operative time was 180 min, with no complications (Figure 3C).

Postoperatively, pain was managed with intravenous paracetamol (1 g every 6 h) and tramadol (50 mg as needed). Prophylactic ceftriaxone (1 g daily) was transitioned to oral amoxicillin-clavulanate (625 mg twice daily for seven days), with enoxaparin (40 mg daily for five days). The drain output decreased from 120 mL on postoperative day (POD) 1 to <50 mL (POD 3), allowing removal. Oral intake began with liquid intake on POD 2, advancing to a soft diet on POD 4. The patient was discharged on POD 5 with ibuprofen (400 mg, as needed) and chlorhexidine rinses, with no dehiscence or infection.

At the one-month follow-up (March 5, 2024), wound healing was complete, with minimal scarring. Intraoral healing post-extraction was satisfactory, with no neurosensory deficits, xerostomia, or dysphagia. Because of the clear margins (>1 mm) and limited nodal involvement (2/28 positive nodes, no ENE), adjuvant radiotherapy was deferred for surveillance. The patient was followed up regularly. The patient was followed up every three months for the first year and then every six months thereafter, with clinical examination and neck ultrasound at each visit and annual MRI for local surveillance. Chest radiographs were obtained yearly to screen for pulmonary metastasis. “Disease-free” status was determined based on the absence of clinical, radiologic, or cytologic evidence of recurrence. At the last follow-up, the patient remained disease-free. Functional outcomes were satisfactory, with normal swallowing and speech, mild transient xerostomia, and intact facial nerve function.

## Discussion

MEC represents 10-15% of salivary gland tumors and one-third of malignancies, with submandibular gland involvement in only 8-13% of cases, underscoring its relative rarity compared with parotid lesions [1,6]. Unlike parotid MEC, submandibular variants exhibit higher malignancy potential, with 20-50% nodal metastasis rates and more aggressive behavior due to their anatomical proximity to neurovascular structures [7,12]. Our case of a 53-year-old man with insidious left submandibular swelling progressing to pain aligns with the reported presentations: painless masses in 70-80% initially, evolving to symptomatic in advanced disease, often mimicking benign sialadenitis or pleomorphic adenoma. Our patient's two-month history without systemic symptoms highlights the indolent onset typical of intermediate-grade tumors, which delayed diagnosis until local extension occurred.

Diagnostic challenges in salivary gland neoplasms are well-documented, with FNAC serving as a cornerstone for preoperative evaluation. FNAC achieves 80-95% sensitivity and 90-98% specificity for salivary tumors, enabling risk stratification and surgical planning [13]. In our case, FNAC's triphasic cellularity (epidermoid, mucous, and intermediate cells) with a mucinous background correctly suggested low-to intermediate-grade MEC. However, MEC's cytological overlap with Warthin tumors or acinic cell carcinoma poses pitfalls, as noted in cases where cellular pleomorphism leads to undergrading [14]. Postoperative IHC definitively confirmed the diagnosis via p63 and CK7, with Ki-67 at 15% affirming intermediate-grade MEC [10]. This multimodal approach mirrors the literature, emphasizing IHC's role in 70-80% of equivocal FNAC cases for salivary malignancies [15].

Surgical management remains the gold standard for MEC, with wide local excision and neck dissection for nodal disease, yielding a five-year overall survival (OS) of 79-84% across grades [7]. In our patient, en bloc resection of the submandibular/sublingual glands with 1.5 cm margins and MRND type III (28 nodes, 2 positive) achieved clear margins without bony invasion, aligning with guidelines for T2N1M0 disease [11]. Tooth extraction (32-35) facilitated access, a practical adjunct in intraoral extensions, as in a reported anterior lingual MEC case where misdiagnosis delayed the intervention. Adjuvant radiotherapy due to low-risk features (no ENE, focal perineural invasion, clear margins >1 mm) provides evidence that intermediate-grade MEC with negative margins requires observation, reducing xerostomia risk while maintaining efficacy [16]. Comparable outcomes are seen in a post-chemotherapy submandibular MEC case, where surgery alone is sufficient for localized disease [17].

Prognostically, intermediate-grade MEC confers 62-97% five-year OS, superior to high-grade (0-43%) but inferior to low-grade (92-100%), influenced by nodal status and margins [5,18]. The submandibular site showed worse outcomes than the parotid site (hazard ratio 1.5-2.0), emphasizing vigilant surveillance. This case underscores FNAC's diagnostic utility of FNAC and the curative potential of surgery for submandibular MEC, advocating multidisciplinary care for optimal functional preservation. MAML2 (CRTC1/3-MAML2) fusion testing, which has prognostic relevance in MEC, was not performed due to limited molecular diagnostic resources. Additionally, p63 rather than p40 was utilized for squamous differentiation due to unavailability of p40 antibody during testing. Long-term follow-up (3-5 years) will monitor for late recurrences, common in 20-30% of intermediate-grade cases. Future studies on advanced molecular markers may refine the grading and therapy.

## Conclusions

The case of a 53-year-old male patient with MEC of the left submandibular and sublingual glands highlights the success of comprehensive diagnosis and surgical management. FNAC, supported by IHC analysis, confirmed the diagnosis and guided the effective surgical resection and neck dissection. The patient achieved a disease-free status at one year with preserved function, emphasizing the importance of tailored surgery and vigilant follow-up for salivary gland malignancies.
